# PAMAM versus PEI complexation for siRNA delivery: interaction with model lipid membranes and cellular uptake

**DOI:** 10.1007/s11095-022-03229-7

**Published:** 2022-03-22

**Authors:** Patrick K. C. Chang, Clive A. Prestidge, Kristen E. Bremmell

**Affiliations:** University of South Australia, Clinical and Health Sciences, Adelaide, South Australia 5000 Australia

**Keywords:** siRNA, dendrimer, PEI, AFM, lipid-bilayer

## Abstract

**Purpose:**

Cationic polymers have many advantages as vectors for mediated cellular entry and delivery of siRNA. However, toxicity related to their cationic charge has compromised clinical use. It is hypothesized that the siRNA-vector complex composition and properties can be controlled to optimize therapeutic performance. Here we investigate siRNA complexes with branched polyethylenimine (bPEI) versus generation 4 polyamidoamine dendrimers (PAMAM) on interactions with immobilized lipid membranes, and cellular uptake and toxicity.

**Methods:**

A model siRNA was complexed with either PAMAM or bPEI, and their size and zeta-potential characterized. Interaction of the complexes and parent polymers with lipid bilayers was investigated using atomic force microscopy and correlated with the uptake and toxicity in HeLa cells.

**Results:**

PAMAM and its siRNA complexes formed circular shaped micron-sized holes in lipid bilayers, while bPEI formed nanoscale holes. Flow cytometry and fluorescence microscopy demonstrated PAMAM-siRNA complexes to have a higher cellular uptake than bPEI-siRNA complexes. bPEI-siRNA complexes did not impact on viability, however PAMAM-siRNA complexes demonstrated increasing cell toxicity as N/P ratio increased. PAMAM-siRNA complexes accumulated around the cell nucleus, while PEI-siRNA complexes were located closer to the cell wall.

**Conclusion:**

Complexation of PAMAM dendrimer or bPEI with siRNA modified physicochemical properties of the parent polymer, however it did not impact on the mechanism of interaction with model lipid bilayers or how the polymer/siRNA complex interacted and was internalized by HeLa cells. Interaction of siRNA polymer complexes with cells is related to the action of the parent polymer.

**Graphical abstract:**

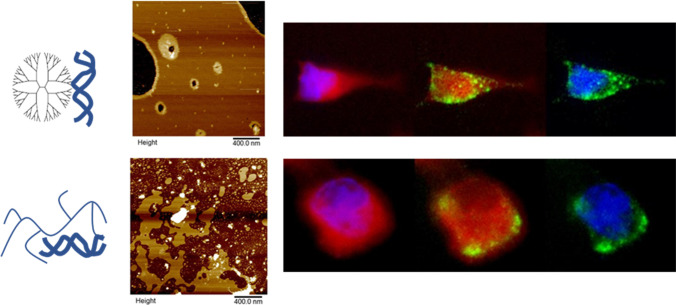

## Introduction

Cationic polymeric vectors represent a class of promising delivery systems for siRNA, implicated for the treatment of many gene-related diseases. The cationic properties of these delivery systems provide suitable characteristics for the complexation of nucleic acids such as siRNA, utilizing mainly electrostatic interaction in the formation of a stable polyplex system (1–4), whilst also facilitating their entry into cells (5–7). Efficient facilitation of siRNA entry through the cell membrane into the cytoplasm is required to allow them to exhibit their biological activity. Therefore, strong membrane surface binding is critical for effective internalization into the cell, which is mainly driven by electrostatic interaction between the delivery systems surface groups and lipid head groups of the cell membrane (6, 8, 9).

Understanding how these cationic polymers and their complexes with siRNA interact with the cell surface membrane is critical in designing optimal delivery systems. Previous studies have reported cationic polymer interaction with supported lipid bilayers (SLBs), representative of cell membranes. Polymers such as polyethylenimine (PEI) and polyamidoamine dendrimers (PAMAM) adsorb to the lipid-bilayer and induce bilayer defects and hole formation in SLBs (10–14). Several studies have demonstrated effects upon modification of these cationic delivery systems surface properties in relation to their behaviour *in-vitro*, i.e. cytotoxicity and transfection efficiency (15, 16). Complexation of PAMAM dendrimer or branched-PEI with siRNA, has been shown to generate particles with altered physicochemical properties to the parent polymer, such as reduced charge and increased size (17). However, how these changes impact on interaction with and internalization into cells has not been well documented.

Despite the potential use of cationic polymers in siRNA delivery, a lack of fundamental understanding of the relationship between the delivery system’s physicochemical properties and *in-vitro* behaviour limits their bio-application as gene delivery systems. This study aims to establish a relationship between the physicochemical properties of polymer-siRNA complexes, their interaction with SLB’s, cellular uptake, internalization pathway and cytotoxicity mediated by two cationic polymers used in gene delivery. Branched polyethylenimine (bPEI) has been used extensively for complexation and delivery of nucleic acids and generation 4 (G4) PAMAM dendrimers with a well-defined branched structure have shown efficient gene delivery. G4 PAMAM dendrimer is a branched polymer with terminal primary amine groups, tertiary amines at each branching point, and a molecular weight of 14 kDa. bPEI is branched cationic polymer containing primary, secondary and tertiary amines with a molecular weight of 25 kDa. Both molecules therefore have a pH dependent cationic nature, with positive charges increasing with a decrease in pH. The primary amines of PAMAM dendrimers are protonated at pH 7 and as the pH decreased to 4, the tertiary amines become charged (18), while for bPEI, modelling has demonstrated that approximately 55% of the amine groups are protonated under physiological conditions (19). A comparison between the parent polymer and their siRNA complexes will detail the influence of complexation on the cell internalization. These studies assist in further understanding of cationic polymer transfection agents and their clinical translation.

## Materials and Methods

### Materials

Generation 4 ethylene diamine (EDA) core polyamidoamine dendrimer in methanol (G4, Mw = 14,214.17 g/mol, 64 amine terminal end groups) (PAMAM), branched polyethylenimine polymers (bPEI, Mw = 25,000 g/mol), Mission® siRNA duplex in lyophilised form (siRNA, Mw = 13,273 g/mol) directed against cyclin-B1 with the following sequence: sense 5′ –CAUGUUUAUUGCAAGCAA[dT][dT] - 3′, and GelRed nucleic acid gel stain (10,000X in water) were acquired from Sigma-Aldrich (Castle Hill, NSW, Australia) and were used without further purification. 4-(2-hydroxyethyl)-1-piperazineethanesulfonic acid (HEPES) in 1 M concentration used as a buffering agent was purchased from Life Technologies (Mulgrave, VIC, Australia). Agarose used for gel electrophoresis, bromothymol blue (sodium salt), EDTA (0.5 M Sterile), glycerol, Tris base were purchased from Astral Scientific (Gymea, NSW, Australia). Milli-Q purified water treated with diethylpyrocarbonate (DEPC) was used for dilution throughout the experiment. Lyophilised 1-palmoyl(d31)-2-oleoyl-sn-glycero-3-phosphocholine (16:0 – 18:1 PC) (POPC), and 1,2-dimyristoyl-sn-glycero-3-phosphocholine (DMPC) was purchased from Avanti Polar Lipids (Alabaster, Alabama, USA). Lyophilised 1,2-dioleoyl-sn-glycero-3-phospho-L-serine (18:1 - 18:1 PS) (DOPS) was obtained from Lipoid (Steinhaussen/ZG, Switzerland). Both lipids were dissolved separately to desired concentration in chloroform.

#### Preparation of polyplexes

50 nmol of siRNA in lyophilised form was first diluted in DEPC treated Milli-Q water to give siRNA aliquots of 100 μL in a RNAse/DNAse free sterile microcentrifuge tubes with a final concentration of 10 μmol/L. Complexation of siRNA with PAMAM dendrimers and bPEI was performed in 10 mM HEPES buffer at pH 7.4, as previously described (1). Briefly, the desired PAMAM or bPEI concentration was prepared with 10 mM HEPES buffer and siRNA solution added drop-wise, followed with 5 s of vortex mixing. The final mixture was left at 25°C overnight to allow the complex to fully form. The polyplexes were prepared at different polymer to siRNA charge ratio (N/P) (calculated as previously described (20)), of 1:1, 2:1, 4:1, 6:1, and 8:1.

#### Particle size and zeta potential

Hydrodynamic diameter (measured by dynamic light scattering (DLS)) and zeta potential were determined using a Malvern Zetasizer Nano-ZS (Malvern Instruments, Worcestershire, UK) equipped with a 4 mW He-Ne laser operating at 633 nm wavelength and detection optics. Each measurement was performed in triplicate in buffer containing 5 mM CaCl_2_, with 30 s delay between each run. All measurements were performed at 25°C.

#### Lipid bilayer formation

Small unilamellar vesicle (SUVs) liposomes for lipid bilayer formation were prepared, as previously described (10). Three lipid bilayer systems were prepared, including neutral DMPC, or POPC, or a mixed lipid system consisting of neutral POPC, and negatively charged DOPS at a ratio of 4:1, yielding a net negative charge. These systems were chosen as mimics for human cell membranes which consist to a large extent of phosphocholine (PC) lipids, making POPC neutral lipids a relevant lipid bilayer model for understanding nanoparticle interaction. The lipids (5 mg/mL) were dissolved in chloroform and mixed in a round bottom flask for 10 – 15 min. Rotary evaporation of the chloroform under a vacuum for 1 h formed a thin lipid film on the surface of the flask, which was then re-hydrated in 20 ml of buffer 1 (10 mM HEPES buffer, 150 mM NaCl, and 5 mM CaCl_2_) for 3 h, yielding a final concentration of 0.25 mg/ml. Upon re-hydration, a turbid solution consisting of large multilamellar vesicles (LMVs) was vortexed prior to undergoing extrusion through 800, 400, 200 and 50 nm polycarbonate membranes 10 times at 27°C using an extruder, used within 1 week of preparation or stored at 4°C when not in use. Particle diameter was measured prior to use.

Model lipid membranes were formed by SUV deposition, following procedures modified from a previously described method (21). Freshly cleaved mica (1 cm^2^) attached to a metal sample puck by double sided tape was prepared by incubating 75 μL of 100 mM CaCl_2_ for 10 min on the substrate. After incubation, CaCl_2_ was removed by rinsing with buffer (10 mM HEPES, 150 mM NaCl). A liposome dispersion (75 μL) in buffer containing 5 mM CaCl_2_ was deposited onto the mica surface. Ca^2+^ was reported to enhance binding and rupture of liposomes on the surface (22). After 30 min, excess lipids were removed by gently rinsing with HEPES buffer (pH 7.4). A second liposome solution was then re-introduced following the process above to ensure full coverage of the mica surface. Formation of each lipid bilayer was confirmed by AFM imaging prior to introducing polymer or polyplexes into the lipid bilayer system. The lipid bilayer was kept hydrated at all times.

#### Zetapotential of SLBs

The zeta potential of SLBs prepared on a 2.5 cm diameter mica substrate was measured using a ZetaSpin Model 1.2 apparatus (ZetaMetrix Inc., USA). This method determines the zeta potential of planar solids using streaming potential (23). HEPES buffer prepared at 10 mM concentration was used as the background electrolyte for solution conductivity. The SLBs were prepared following the procedure detailed above. Details of the apparatus and method are as described previously (23).

#### Atomic Force Microscopy (AFM)

AFM imaging was performed on SLBs, and the subsequent impact that bPEI, PAMAM and their siRNA complexes have on the SLBs in-situ (in liquid) using a Multimode 8 (Bruker, USA) in ScanAsyst mode, equipped with a liquid cell (DI) and a silicon nitride cantilever with nominal tip radius of 2 nm (SCANASYST-FLUID+, Bruker, USA). The resonance frequency was set between 100 and 200 kHz, with spring constant between 0.35 – 1.4 N/m. Scan rate employed during imaging range from 0.977 – 0.501 Hz depending on the roughness of the image. Images were taken at high resolution of 512 × 512. After taking an initial image of the SLB, 75 μL of polymer or polyplex solution was pipetted onto the surface and incubated for 5 min. The substrate surface was then gently rinsed with 10 mM HEPES buffer before another image was obtained and analysed with nanoscope analysis V1.5. All images were performed in buffer (24).

#### FITC labelling of PAMAM dendrimer, bPEI polymers and their siRNA complexes

PAMAM and bPEI polymers were separately conjugated with FITC fluorophores to afford green-labelled polymers. Conjugation was carried out via amide bond between primary amine groups on dendrimer/bPEI polymers to succinimidyl ester on fluorophore. FITC was firstly predissolved in acetone to obtain a final concentration of 1 mg/ml. Unlabeled polymer solutions (1000 nmol/L, 100 nmol/L) were dissolved in 10 mM HEPES buffer at pH 7.4 and added to the FITC dye drop-wise while gently mixing at a molar ratio of 1:2. Fluorescein labelled polymer solutions were then allowed to stand overnight in a dark place at room temperature. Polymer-siRNA complexes were firstly prepared and equilibrated overnight prior to FITC conjugation.

#### Cell Culture

Human epithelial cervix carcinoma cells (HeLa) were maintained in DMEM cell culture medium supplemented with 10% fetal bovine serum and 1% penicillin-streptomycin at 37°C in a 5% CO_2_ humidified atmosphere. Cell lines were subcultured every 3-4 days and harvested from sub-confluent culture (70 – 80%) using 1x trypsin-EDTA solution.

#### Flow Cytometry

Cellular uptake of polymer and complexes were analysed using flow cytometry (Beckman Coulter, Brea, CA, USA) and data analysed using Kaluza V1.2 software (Beckman-Coulter). After 1 h treatment with either of the polymers or their siRNA complexes, the cells were detached using trypsin-EDTA 1x and were washed with cold PBS. Cells were centrifuged at 1200 RPM for 5 min to obtain a cell pellet, which was washed twice before resuspending in PBS for analysis. Internalized polymers and their complexes with siRNA were differentiated from membrane-bound particles by the trypan blue fluorescence quench method. Briefly, cells were treated in duplicate, with trypan blue (1.2 mg/ml) added to every second sample and allowed to incubate for 10 min prior to analysis (25). Counts were gated at 10,000 events and only viable cells were analysed.

#### Cytotoxicity studies

Cytotoxicity of polymers/complexes was studied in opti-MEM (reduced serum) using XTT (Thermo Fisher, VIC Australia) assay. The assay was performed in triplicate (three separate wells) with cells seeded at 5000 cells/well in a 96-well plate using DMEM culture medium containing 10% FBS and 1% P/S. Cells were then incubated for 24 h at 37°C with 5% CO_2_ atmosphere. After incubation, serum medium was removed, and cells were treated with opti-MEM. Varying concentration of cationic polymers (1 nmol, 10 nmol, and 100 nmol) and charge ratio (N/P) complexes at siRNA concentration of 25 nmol were then added to separate wells. Treated cells were incubated and cytotoxicity assessed after 24 and 48 h.

#### Fluorescence microscopy

HeLa cells were seeded at 5000 cells/cm^2^ in serum growth media onto a permanox 8-well chamber slides (Thermo Fisher, VIC, Australia), cultured under normal conditions in an incubator overnight. After 24 h incubation, medium was replaced with reduced serum media (opti-MEM) and equilibrated for 20 min. Cells were then treated with either one of the polymers or siRNA complexes (equivalent to nmol siRNA) conjugated with FITC fluorophores for 1 h at 37°C (as previously undertaken (26, 28)), after which the cells were washed three times with PBS to remove unbounded polymers/complexes. Cells were fixed with 4% para-formaldehyde for 20 min at room temperature, and later washed with PBS. Plasma red cell mask stain was then added to each well and incubated for an additional 20 min. Mountant was added prior to being covered with a glass coverslip, which was sealed and imaged immediately using an Olympus BX53 fluorescence microscope with Cellsens dimensions image software (Olympus Imaging Australia, NSW, Australia).

#### Statistics

Statistical analysis was performed using GraphPad Prism (version 7.00, GraphPad Software, Inc., San Diego, California). Difference between samples were analysed using student un-paired T-test with Welch’s correction assuming both samples do not have equal standard deviations. P value >0.05 was statistically insignificant, whilst P < 0.001 was considered highly significant.

## Results

### Preparation and characterization of siRNA complexes

The diameter of PAMAM-siRNA and bPEI-siRNA complexes were 260 to 300 nm and 160 to 170 nm, respectively, independent of N/P ratio, as reported previously (17). Both the PAMAM-siRNA and bPEI-siRNA complexes were positively charged, in the range of +10 to 30 mV and + 30 to 50 mV, respectively (17). Therefore, all polymer-siRNA complexes demonstrated an increase in size compared to the polymeric vector, and due to their positive charge were expected to have a strong affinity for negatively charged cell surfaces.

### SLB formation and characterisation

SLBs composed of various mixtures of DMPC, POPC and DOPS were prepared via liposome deposition on mica. Size and zeta potential data for the liposomes and corresponding SLBs are provided in Table 1.

**Table 1:** Hydrodynamic size and zeta potential of liposomes in aqueous solution measured using Dynamic Light Scattering (DLS) with buffer containing 5 mM CaCl_2_. Zeta potential of SLBs measured using Zetaspin.

	Lipid Bilayer Zeta Potential (mV)	Liposome Zeta Potential (mV)	Hydrodynamic Size (nm)
Mica Surface	−102.9 ± 7.2		
Mica Surface (CaCl_2_ modified)	−44.3 ± 6.0		
4:1 POPC:DOPS	−30.8 ± 1.7	−9.3 ± 0.2	53.3 ± 20.6
POPC	−23.3 ± 3.6	5.0 ± 0.1	52.75 ± 16.3
DMPC	−24.9 ± 5.8	4.4 ± 0.3	122.80 ± 5.4

**Fig. 1:**
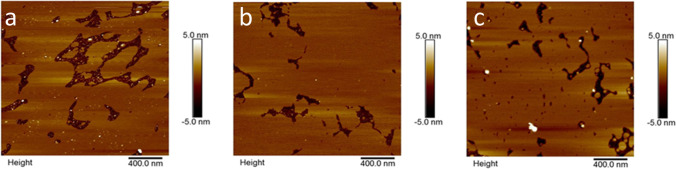
AFM images of the lipid bilayer surfaces (a) 4:1 POPC:DOPS, (b) POPC and (c) DMPCs

Liposomes prepared from the neutral POPC and DMPC lipids displayed a slightly positive charge of 5.0 ± 0.1 mV and 4.4 ± 0.3 mV, respectively, while 4:1 POPC:DOPS liposomes displayed a negative potential of −9.3 ± 0.2 mV. The zeta potential of mica was found to reduce from −102.9 to −44.3 mV, as a result of Ca^2+^ adsorption. SLBs formed from the negatively charged 4:1 POPC:DOPS system further reduced the overall surface potential to −30.8 mV. Neutral lipid bilayer systems showed a further reduction of overall surface charge to −23.3 and − 24.9 mV corresponding to POPC and DMPC bilayers, respectively. Therefore, the mixed lipid system (4:1 POPC:DOPS) was shown to retain the negatively charge characteristics and representative of a target cell surface. The overall surface charge of neutral bilayer systems (POPC and DMPC) deposited on mica did not reach 0 mV, possibly as a result of defects in the SLB. AFM images of the SLBs (Fig. 1) exhibited a smooth and flat surface characteristic of a lipid bilayer with some defects in the form of nano-scale holes and gaps in between the monolayers. Defects were found to be in the height of approximately 4 – 5 nm, indicating successful formation of an SLB, commonly observed for lipid bilayers (22).

### Interaction with SLBs

To investigate the interaction of cationic polymers with cells, SLBs have been utilized as model cell membranes. A major component of cell membranes are phospholipids, in addition to cholesterol and proteins. Representative of a cell membrane, lipids with a phosphocholine (PC) head group are commonly employed. The bilayer fluidity can be controlled through saturation of the lipid acyl chains. SLBs prepared from lipids with unsaturated acyl chains (such as POPC) have a low transition temperature and a fluid-like nature, compared to DMPC with saturated acyl chains that form a gel phase lipid bilayer. Cell membranes are often negatively charged, with addition of lipids containing a phosphotidylserine (PS) headgroup (DOPS) providing this negative charge. Through studying a range of SLBs, cell membrane properties such as charge and fluidity can be modelled.

**Fig. 2:**
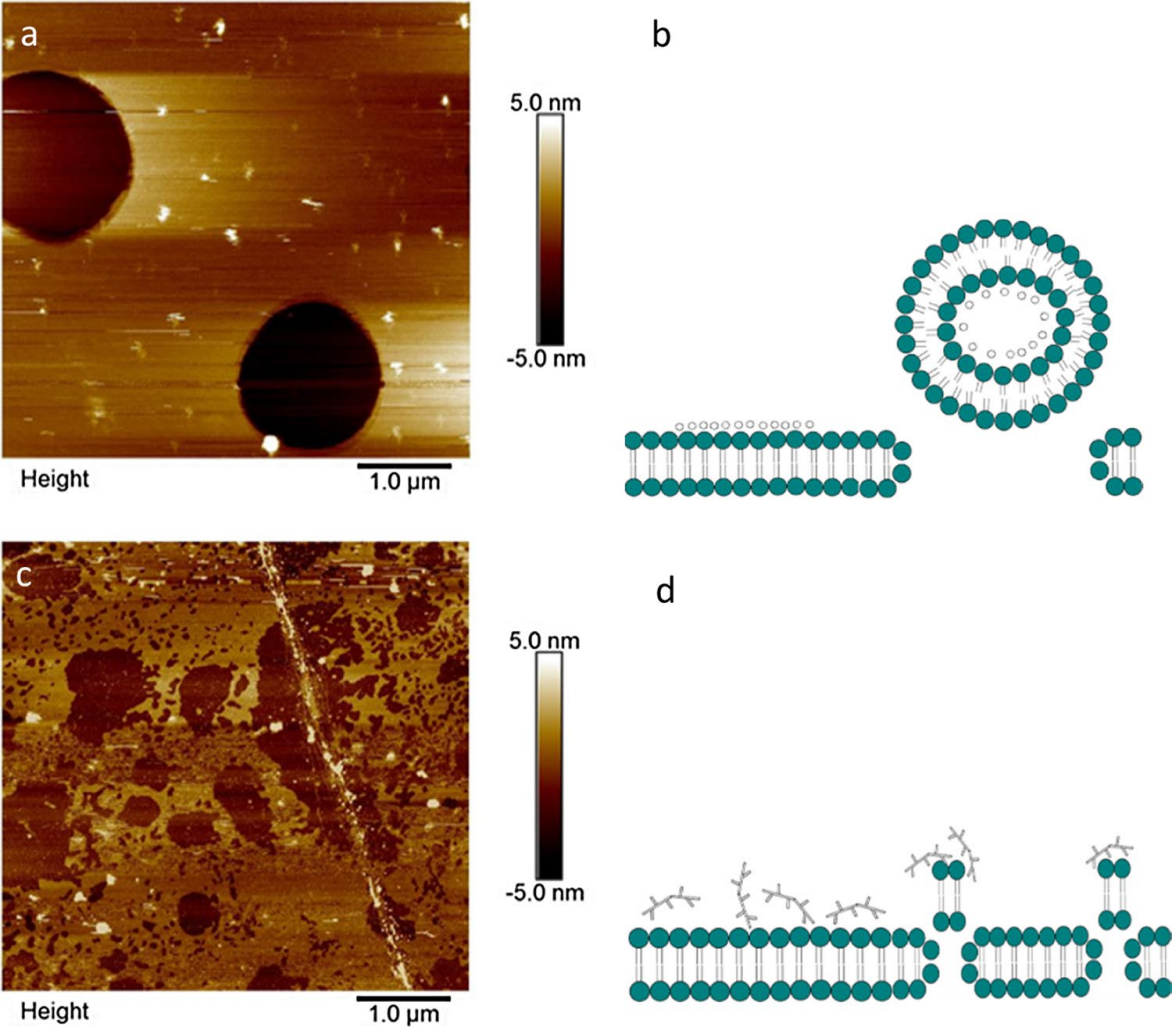
AFM images of POPC:DOPS SLB system after 5 min contact with (a) PAMAM and (c) bPEI polymer. Schematic illustration of removal mechanism of (b) PAMAM and (d) bPEI

PAMAM interaction with a negatively charged mixed SLB (4:1 POPC:DOPS) deposited on mica is shown in Fig. 2a. When dendrimers were exposed to the bilayer surface, there is evidence they adsorbed as small aggregates (Fig. 2a) on top of the bilayer. Additionally, they demonstrate the ability to disrupt the integrity of bilayer via formation of circular holes in lipid bilayers. An illustration of this mechanism has been depicted in Fig. 2b. Similar behaviour was observed upon exposure of PAMAM onto both neutral lipid systems, POPC and DMPC (data not shown), also correlating with previous reports (10, 11).

Interaction of bPEI with a mixed SLB was investigated to elucidate their destabilization mechanism (Fig. 2c) after 5 min exposure; disruption of lipid bilayer integrity was observed. bPEI was shown to induce removal of lipid from the surface by formation of nano-scale holes on areas of lipid bilayer surface that were intact, as depicted in Fig. 2c. Removal of lipids from pre-existing defects was also observed, with similar destabilization noted upon interaction with the two different neutral lipid bilayer systems (data not shown). No visible particles resembling bPEI polymers were observed on the surface. This suggests the bPEI polymers were removed along with the lipid bilayer upon rinsing of buffer across the bilayer surface to remove excess particles that may interrupt the imaging process. A schematic illustration of bPEI polymer removal mechanism is provided in Fig. 2d.

**Fig. 3:**
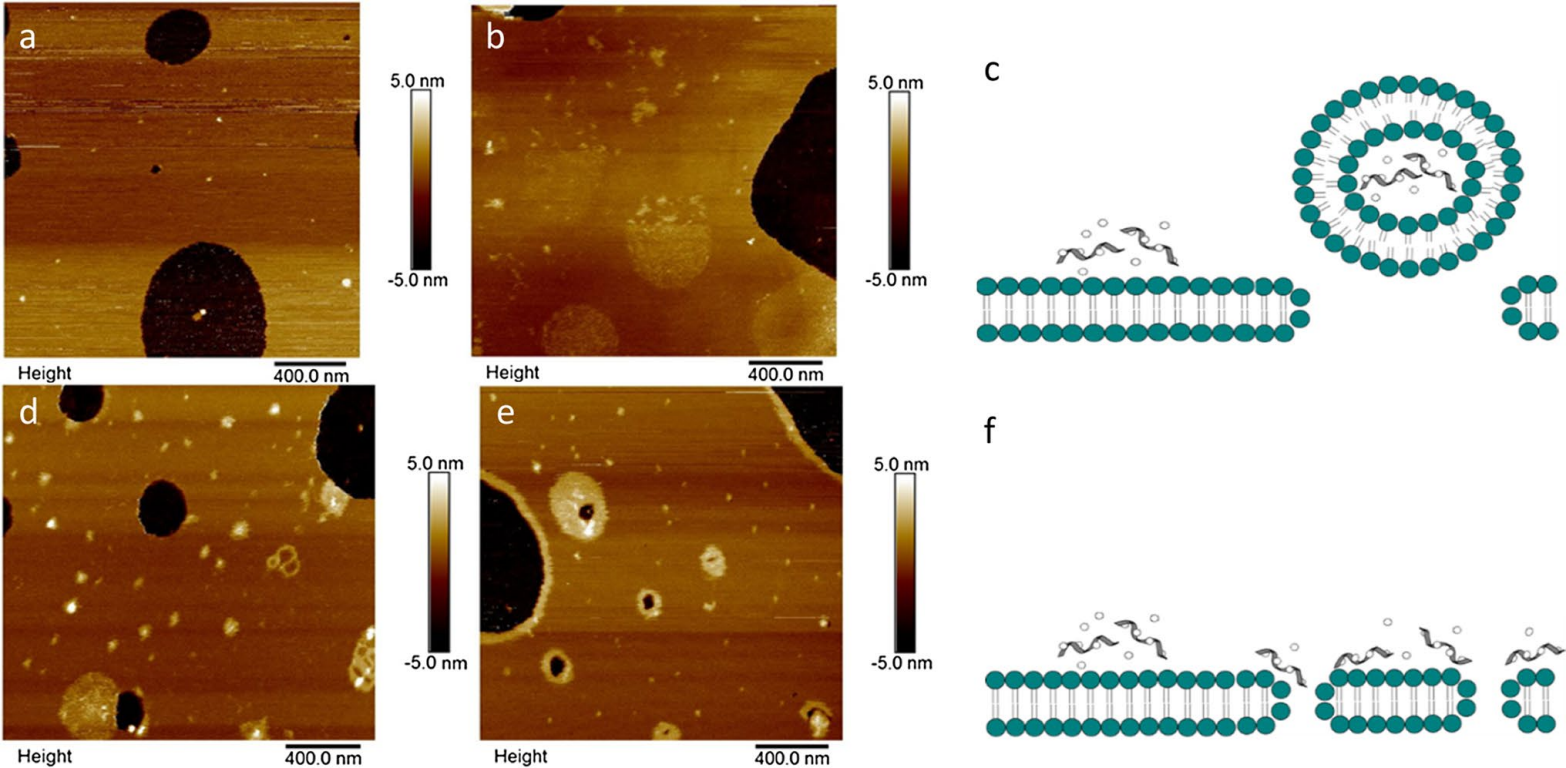
AFM images of PAMAM-siRNA complex interaction with SLBs. (a) N/P 2:1 on mixed 4:1 POPC:DOPS SLB, (b) N/P 2:1 on POPC SLB, (c) schematic showing PAMAM-siRNA complexes removing part of the lipid bilayer, (d) N/P 4:1 exhibiting similar behaviour on POPC lipid bilayer, (e) complex of N/P 6:1 on DMPC SLB, and (f) schematic of PAMAM-siRNA complex adsorbing to the SLB

The influence of PAMAM-siRNA complexes on the integrity of three different SLBs was visualized by AFM (Fig. 3). Formation of circular shaped holes were observed at all charge ratio (N/P) PAMAM-siRNA complexes upon exposure to all 3 SLBs. As the N/P ratio increased, an increased number of adsorbed polyplexes were observed (Fig. 3d and e). The presence of aggregation indicated by increased height profile around formed defects and on the surface of intact lipid bilayer implies that these aggregates may play a role in the formation of circular holes. PAMAM-siRNA polyplexes exhibited similar destabilization mechanism to PAMAM, whereby AFM analysis showed adsorption of monomeric particles and disruption by formation of circular shaped holes. The different characteristics of SLBs did not influence the way these complexes interact with the lipid bilayer. Schematic representation of dendrimer-siRNA complex adsorption and removal mechanism at the lipid bilayer interface is depicted in Fig. 3c and f.

**Fig. 4:**
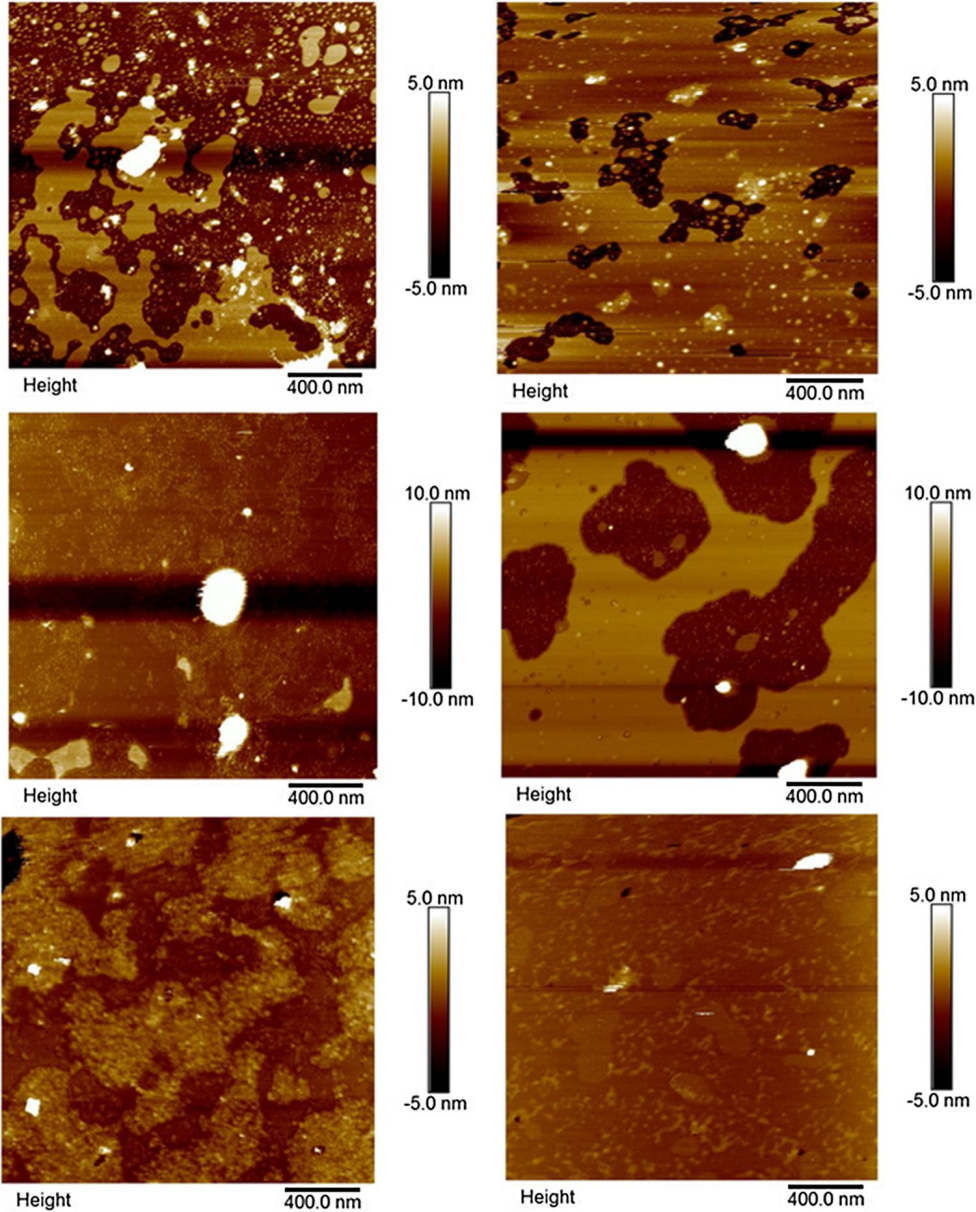
AFM images of bPEI-siRNA complexes on SLBs, (a) N/P 4:1, and (b) N/P 2:1 on 4:1 POPC:DOPS SLBs. (c) N/P 4:1 and (d) N/P 1:1 on POPC SLB. (e) High charge ratio (N/P) 8:1 and (f) N/P 2:1 on DMPC lipid bilayer

The adsorption and destabilization mechanism of bPEI-siRNA complexes on SLBs was investigated (Fig. 4). High charge ratio (N/P > 4:1) bPEI-siRNA complexes demonstrated significant removal of lipids from negatively charged 4:1 POPC:DOPS SLBs, as displayed in Fig. 4a. Pre-existing intact lipid bilayers were also removed and complexes can be seen to adsorb onto the exposed mica substrate, represented by small spherical particles. On the other hand, lower charge ratio (N/P < 2:1) complexes displayed little removal of intact SLBs (Fig. 4b), with adsorption of spherical complex particles observed on the lipid surface. On neutral POPC lipid bilayer, high charge ratio bPEI-siRNA complexes (N/P > 4:1) demonstrated significant removal as depicted in Fig. 4c and lower charge ratio (N/P 1:1) complexes exhibited little removal of lipids from this surface (Fig. 4d). On the other hand, complexes exhibit different behaviour on neutral DMPC lipid bilayer; high charge ratio (N/P) 8:1 bPEI complexes displayed little or no removal from DMPC bilayers (Fig. 4e), and accumulation of aggregated particles can be seen around the bilayer surface. Furthermore, lower charge ratio N/P 2:1 complexes displayed no lipid removal on DMPC lipid bilayer, as shown in Fig. 4f.

### Cellular uptake and toxicity

**Fig. 5:**
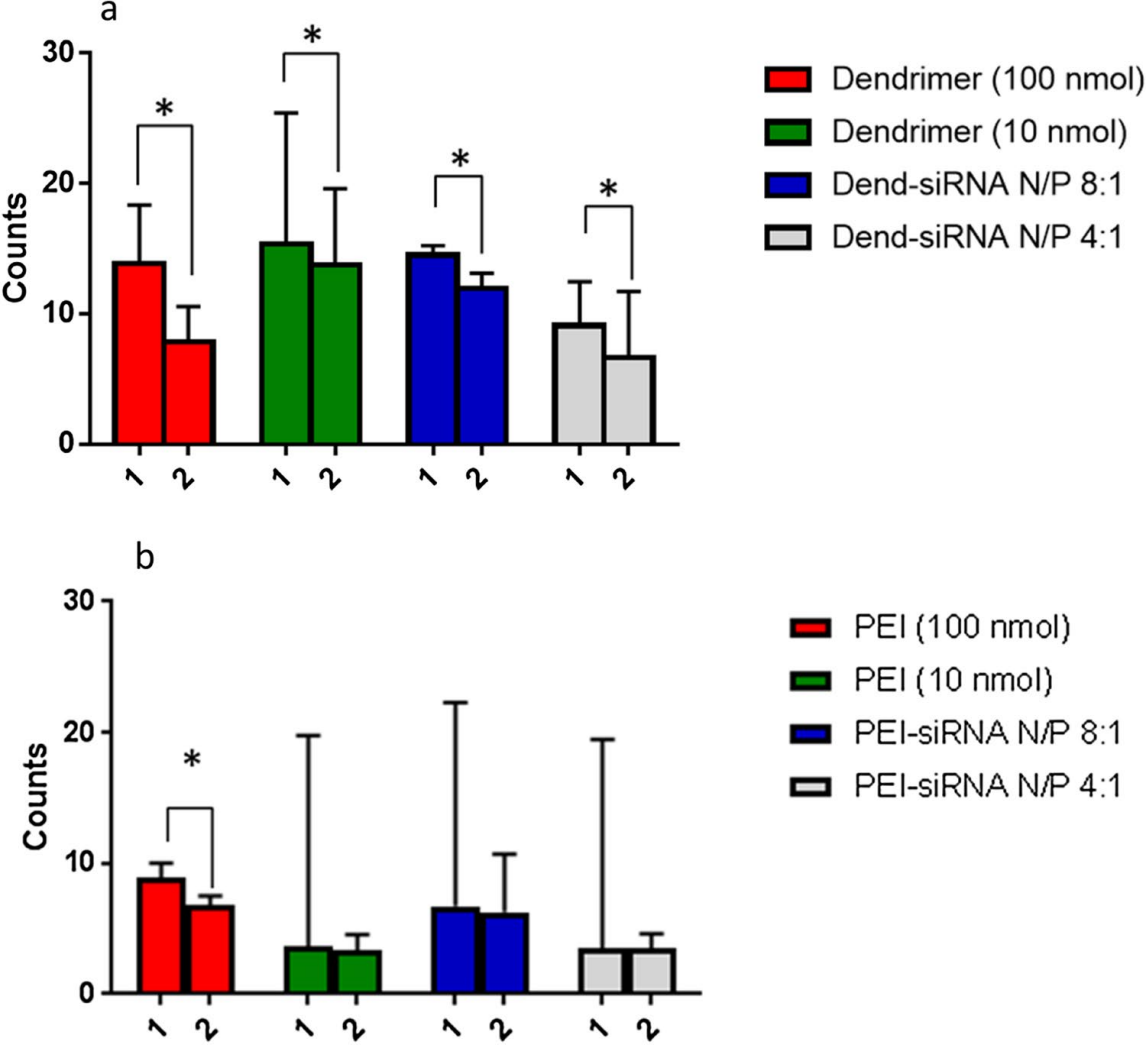
Flow cytometry of FITC labelled (a) PAMAM dendrimers and their siRNA complexes and (b) FITC labelled bPEI and their siRNA complexes into Hela cells. Quantitative measurements of fluorescence performed in duplicates treated with either (1) PBS or (2) trypan blue as a means to quench extracellular conjugated FITC fluorescence incubated for 1 h at 37°C. Asterisk (*) indicates statistically significant with a P value <0.05 for comparison between the absence/presence of trypan blue treatments

Flow cytometry investigated the uptake efficiency and role of membrane adsorption in the internalization of PAMAM, bPEI and their siRNA complexes into HeLa cells. PAMAM dendrimers were readily internalized into the cell, independent of PAMAM concentration and complexation with siRNA (Fig. 5). Post-quenching with trypan blue resulted in a significant difference in quantitative measurement of fluorescence in comparison to pre-quenched cells, indicating a difference in the number of membrane bound and internalized PAMAM and their siRNA complexes. The observed quantitative difference indicates a dependency on membrane adsorption for the internalization of both PAMAM and their siRNA complexes. This behaviour has been commonly observed with other polymeric delivery systems that were shown to be successfully internalized following adsorptive endocytosis.

bPEI and their siRNA complexes showed an overall lower amount of quantitative fluorescence in comparison to PAMAM systems (Fig. 5b). This result suggests that bPEI has a lower internalization efficiency in comparison to PAMAM dendrimers, as supported by a comparative study by Seib *et al*. (26), where they found that PAMAM had a higher maximum uptake in comparison to bPEI. After quenching with trypan blue no significant difference between quantitative measurements upon transfection with bPEI performed at low concentrations (10 nmol) and bPEI-siRNA complexes (N/P 4:1 and 8:1) was observed. This indicates that low concentration bPEI and their siRNA complexes are less dependent on membrane adsorption for cellular uptake. However, at a higher concentration of bPEI (100 nmol), a significant difference was measured between quantitative fluorescence measurements before and after trypan blue treatment. Overall, this implies that internalization of bPEI systems depended less on membrane adsorption for cellular uptake, which is consistent with other studies that reported a small decrease in fluorescence quenching occurs as a result of the rapid transport of such polymers across the cell membrane using either paracellular or transcellular pathways (15).

**Fig. 6:**
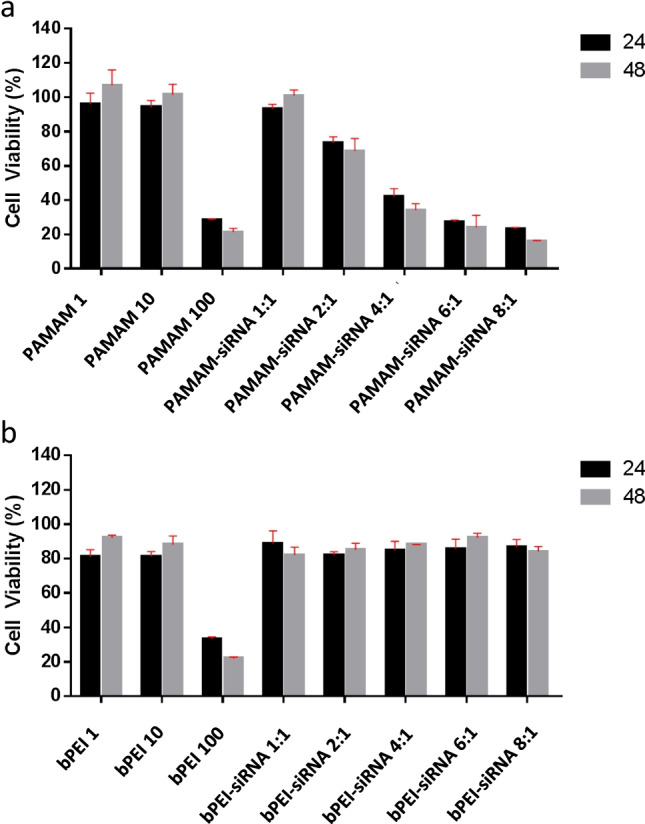
Cytotoxicity of (a) PAMAM and (b) bPEI and their siRNA (25 nmol) complexes on HeLa cells after 24 and 48 h incubation using the XTT assay (n = 3). Polymer concentrations were 1, 10, and 100 nmol, and when complexed with siRNA at N/P ratios of 1:1, 2:1, 4:1, 6:1 and 8:1, were 16, 32, 65.5, 98, 131 nmol respectively for PAMAM and 1.8, 3.6, 7.3, 10.9, 14.5 nmol respectively for bPEI

Cell viability data is provided in Fig. 6. Toxicity of HeLa cells caused by PAMAM and bPEI was concentration dependent, with significant reduction in cell viability being observed for both polymers at 100 nmol, whilst cell viability was shown to remain unaffected upon transfection with lower concentration of both polymers (1 and 10 nmol) with little to no additional cytotoxic effects observed after 48 h (Fig. 6). PAMAM-siRNA complexes showed concentration dependent cytotoxicity (Table 2), with increasing toxicity observed at a charge ratio of 4:1 and above. Data in Table 2demonstrates that cell viability with PAMAM-siRNA complexes is related to the concentration of polymer applied; systems with PAMAM concentrations above 30 nmol show cytotoxicity and siRNA complex systems with 98 and 131 nmol PAMAM result in similar cell viability to that of 100 nmol PAMAM alone. However, bPEI-siRNA complexes showed little toxicity for all N/P ratios tested. This may be attributed to the bPEI concentration within the complexes, which is within the range of bPEI concentrations where cell viability was not impacted, as shown in Fig. 6.

**Table 2:** Size, zeta potential and cell viability (24 h) of the siRNA complexes and the corresponding polymer concentration applied in the HeLa cell cytotoxicity studies, including the standard deviation.

siRNA complex (N/P)	Size (nm)	Zeta potential (mV)	Cell viability (%)	Polymer concentration (nmol)
**PAMAM**				
**1:1**	255 ± 19	18.6 ± 2.5	94 ± 2	16
**2:1**	330 ± 50	10 ± 2	75 ± 3	32
**4:1**	235 ± 36	18 ± 2	43 ± 4	65.5
**6:1**	287 ± 53	28 ± 2	29 ± 0.3	98
**8:1**	270 ± 30	28 ± 1	25 ± 0.3	131
**bPEI**				
**1:1**	167 ± 4	36 ± 2	90 ± 6	1.8
**2:1**	170 ± 10	32 ± 3.5	83 ± 1	3.6
**4:1**	164 ± 7	36 ± 27.5	85 ± 5	7.3
**6:1**	166 ± 3	33 ± 3	86 ± 5	10.9
**8:1**	162 ± 7	38 ± 10	87 ± 4	14.5

### Intracellular imaging

**Fig. 7:**
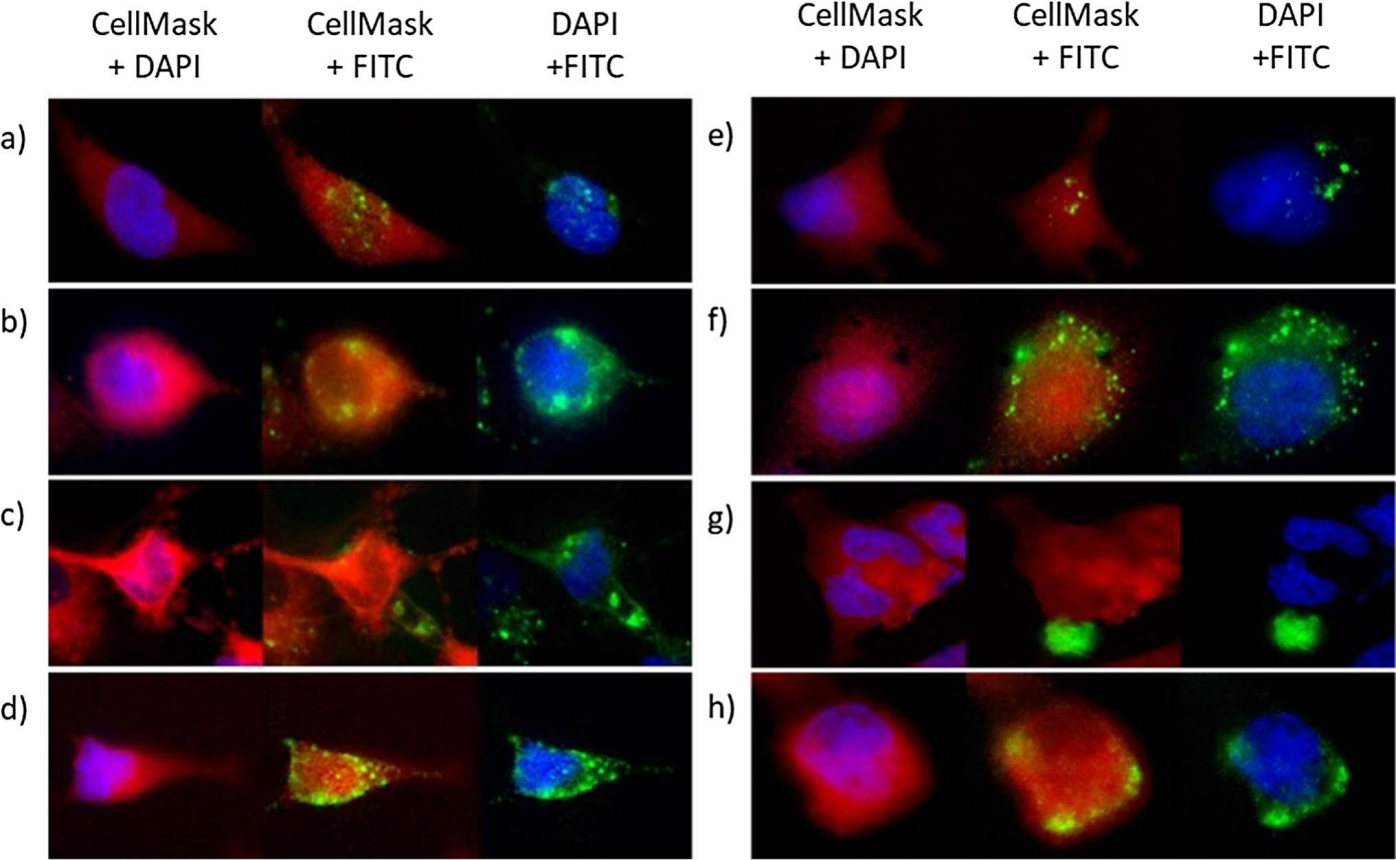
Cellular internalization analysed by fluorescence microscopy of PAMAM dendrimer at (a) 10 nmol (b) 100 nmol, and PAMAM-siRNA complexes at (c) N/P 4:1 (65 nmol PAMAM) (d) N/P 8:1 (131 nmol PAMAM), bPEI at (e) 10 nmol, (f) 100 nmol and bPEI-siRNA complexes at charge ratio (g) N/P 4:1 (7.3 nmol bPEI) and (h) N/P 8:1 (14.5 nmol bPEI) into HeLa cells incubated at 37°c for 1 h. Cell membrane was stained with Red CellMask stain, blue represents the nucleus stained with DAPI, and green represents PAMAM or bPEI complexes conjugated with FITC fluorophore

Epifluorescence microscopy was utilized to visually determine the localization, hence intracellular trafficking of PAMAM, bPEI and their siRNA complexes internalized in HeLa cells (Fig. 7). As shown in Fig. 7a, b, PAMAM demonstrated a high internalization efficiency indicated by the extent of fluorescence present within the cell. In conjunction, cells transfected with lower concentrations (10 nmol) of PAMAM (Fig. 7a) demonstrated lower amounts of fluorescence within the cell. Interestingly, these cationic polymers were observed to accumulate around the perinuclear region, demonstrated by an increased amount of fluorescence around the blue stained nucleus. Such behaviour has been reported for other generation PAMAM dendrimers in various cell lines (27, 28).

FITC labelled PAMAM-siRNA complexes were transfected into HeLa cells at charge ratios of N/P 4:1 and 8:1. High charge ratio (N/P 8:1) complexes were effectively internalized into the cytoplasm (see Fig. 7d). The bright green spots visible in the cell may indicate entrapment of complexes in vesicles. The trafficking of complexes may change over an extended period of time, as observed previously where migration of PAMAM in HeLa cells occurred over 12 h (29). Lower charge ratio (N/P 4:1) PAMAM-siRNA complexes (Fig. 7c) displayed decreased internalization efficiency (lower fluorescence in the cytoplasm). This result correlates with the internalization of PAMAM showing a concentration dependent relationship associated with the internalization of these siRNA complexes mediated by PAMAM. Additionally, complexes at N/P 4:1 and 8:1 displayed similar intracellular trafficking, where they accumulate around the vicinity of the nucleus indicated by high fluorescence around the blue-stained nucleus. Therefore, it has been demonstrated that PAMAM and their siRNA complexes possessed high internalization efficiency and the ability to be successfully trafficked to the perinuclear region in HeLa cells, independent of siRNA complexation.

Fluorescence imaging of cells treated with FITC labelled bPEI (10 and 100 nmol) and siRNA complexes at charge ratio of N/P 4:1 and 8:1, is shown in Fig. 7. At 10 nmol bPEI, little fluorescence was observed in the cells. However, distribution of green fluorescence near the membrane was observed with the transfection of 100 nmol bPEI polymers, indicating effective internalization. However, fewer polymers were observed to be trafficked to the perinuclear region compared to PAMAM, represented by uniform distribution of fluorescence within the cytoplasm.

For the transfection of bPEI-siRNA complexes into HeLa cells, successful internalization was only observed with high charge ratio (N/P 8:1) complexes. This was represented by high amounts of fluorescence within the cell that were in close proximity to the cell membrane, as shown in Fig. 7h. However, cells transfected with bPEI-siRNA complexes of N/P 4:1 (Fig. 7g) displayed little to no fluorescence inside the cell.

## Discussion

PAMAM dendrimer and bPEI, both highly charged cationic polymers, induced destabilization within minutes of exposure to SLBs. AFM imaging demonstrated PAMAM to induce distinctive circular shaped holes, as illustrated actually and schematically in Figs. 2 and 3. Similar findings have been reported in several other studies (10, 11, 30). Adsorption of the cationic polymer systems disrupts the integrity of SLBs by interacting with the zwitterion phospholipid bilayer. PAMAM can act as a bridge between regions of lipid bilayer and this interaction is mainly electrostatic. The strong electrostatic interaction between the polymers’ positively charged terminal group with phospholipid head group results in acquiring sufficient energy for the lipid to bend and coat around the cationic PAMAM dendrimers, previously referred to as ‘dendrisomes’ (31), which are aggregates of dendrimers/complexes with the lipid bilayer via coating of polymer molecules by a lipid membrane. This energetically unfavourable process to maintain vesicle curvature led to removal of lipid from the surface (31–33). Similar lipid bilayer destabilization (complex adsorption leading to formation of circular holes) was observed with N/P ratios from 1:1 to 8:1 PAMAM-siRNA upon exposure to SLBs. Therefore, while complexation with siRNA increases the size and reduces the zeta potential of PAMAM, the mechanism of SLB destabilization was unchanged.

Adsorptive endocytosis requires prior adsorption on the membrane surface, followed by membrane wrapping that promotes encapsulation of adsorbed particles prior to being trafficked into the cytoplasm (34). Flow cytometry analysis revealed that cellular uptake of both PAMAM and PAMAM-siRNA complexes was driven by cell membrane adsorption. This correlated with AFM imaging where adsorption of PAMAM-siRNA complexes was observed. Combined, these results support the idea that internalization of PAMAM and their siRNA complexes are dependent on membrane adsorption for successful uptake by HeLa cells, following an adsorptive endocytosis pathway. Several other studies have shown that dendrimers are taken up through an adsorptive endocytosis process (26, 28, 35). Binding of siRNA to PAMAM has been shown to occur with little change to the dendrimer size and to result in exposed patches of both molecules (1). This conformation may then lead to aggregation of complexes and interaction with SLB and cell surfaces through electrostatic interaction.

bPEI polymer and its complexes were shown to exhibit a different adsorption profile and destabilization mechanism in comparison to PAMAM systems. Schematic representation of bPEI systems destabilization mechanism is shown in Fig. 2, demonstrating their ability to form nano-scale holes on intact lipid bilayer while also removing from edges of pre-existing defects. Phase behaviour of the SLB also influenced their interaction with the delivery system. An example is demonstrated by AFM analysis of varying charge ratio (N/P) bPEI-siRNA complex interaction with a DMPC lipid bilayer, as shown in Fig. 4. AFM analysis depicts no removal mechanism by the highest charge ratio (N/P 8:1) bPEI-siRNA complexes upon adsorption to DMPC SLBs, whilst removal was observed on the other two neutral bilayer systems, more representative of a cell membrane. DMPC lipids exist in several structural states; one is a high temperature fluid phase while other is a low temperature gel phase (36). A study of dendrimer adsorption on lipid bilayer at different phases revealed stronger interaction with higher propensity to destabilize by membrane pore formation and interaction in the fluid phase (above transition temperature) (37). Further, a modelling study concluded that PEI-siRNA complexes interacted more strongly with a negatively charged SLB compared to a neutral one (38). This further suggests correlation between the physicochemical properties of both delivery system and electrostatic interaction with the lipid bilayer system, with integrity of SLBs playing an important role.

AFM analysis revealed increased lipid removal by bPEI-siRNA complexes at higher charge ratio (N/P). However, there was negligible removal exhibited upon addition of low charge ratio (N/P < 2:1) complexes onto mixed and POPC lipid surfaces. A previous study investigated adsorption of bPEI-siRNA complexes on mica; at low N/P ratio adsorption of monomeric complexes was observed, while at high N/P ratio, the bPEI-siRNA complexes formed a network structure across the interface (17). Combining these observations, led to the possibility that bPEI-siRNA complexes do not play a role in the removal mechanism, however lipid removal is the consequence of increasing free bPEI polymer present in solution at higher N/P ratio. Moreover, other studies have demonstrated the effect of PEI physicochemical properties such as shape can influence their cytotoxic effects. Branched PEI was shown to be cytotoxic with cell viability assays demonstrating concentration dependent toxicity effects, whilst linear PEI displayed less toxicity upon exposure at similar concentrations. Their difference in toxicity was attributed to differences in electrostatic interaction exhibited by branched PEI in contrast to linear PEI polymer in correlation with their different surface charge properties (26, 39).

Increased interaction of bPEI-siRNA complexes with SLBs at higher N/P ratio correlates with greater cellular internalization at higher N/P (Figs. 5 and 7). Previous studies investigated the influence of free PEI polymers in solution on gene transfection via PEI-siRNA complexes, and described increasing toxicity in the presence of free PEI polymer in solution (40). Further, purification of free PEI polymer from the polyplex solution, significantly reduced internalization efficiency and toxicity effects (41). Therefore, this clearly highlights the important role of free PEI polymer in solution in causing both destabilization of the lipid bilayer and internalization of polyplexes into biological cells. These findings further support the proposed internalization behaviour of bPEI and their siRNA complexes of being less dependent on the endocytosis pathway as means of entry into cells.

Higher cellular uptake of PAMAM-siRNA was observed from flow cytometry (Fig. 5) and microscopy (Fig. 7). Fluorescence from the FITC labelled polymers was visible for all polymer and complexes investigated, with some fluorescence appearing to reside in intracellular vesicles represented by bright fluorescent dots, suggesting they have not escaped from the endosome after 1 h (42). However, for cationic polymers such as bPEI and PAMAM, the proton sponge effect, as recently reviewed (43), has been proposed to explain endosome escape. Both these polymers contain amine groups considered to neutralize endosome acidification, which leads to osmotic swelling of the endosome and rupture, releasing the complex into the cytosol. Many authors do not support this hypothesis (for example Xu (43), and Vaidyanathan *et al*. (44),) and have proposed other mechanisms, such as increased permeability of the endosome due to free polymeric vectors (44). Further studies would be required to elucidate the endosome escape process and precise mechanisms in action for specific complexes.

## Conclusion

This study has provided further insight into the internalization mechanism of siRNA complexes mediated by G4 PAMAM dendrimers and 25 k branched-PEI polymers, through observation of their interaction with SLBs and cellular studies. Complexation with siRNA altered the size and zeta potential of the PAMAM-siRNA complexes, however the distinctive formation of circular holes in the SLB was found to be independent of the complexation ratio. bPEI and its siRNA complexes were found to generate nano-scale holes on intact bilayer surfaces in addition to removal from pre-existing defects, which was also independent of complexation with siRNA.

G4 PAMAM dendrimers demonstrated a higher internalization efficiency and cytotoxicity effects in comparison to bPEI, independent of complexation with a siRNA. Cellular internalization and cytotoxicity increased with the polymer concentration as N/P ratio increased, irrespective of the altered physicochemical properties after siRNA complexation. For bPEI-siRNA complexes, a high concentration of the cationic polymer was found to improve the overall cellular uptake of these systems. Here, the internalization behaviour of two cationic polymeric systems intended for siRNA delivery was evaluated and represent how differences in polymers physicochemical properties can affect their respective *in-vitro* performance.
